# 2nd NMD4C basic research summer school on promoting standardized protocols to advance translational research in neuromuscular disorders

**DOI:** 10.1177/22143602251408154

**Published:** 2025-12-19

**Authors:** Adrien Rihoux, Emma Sutton, Jaryeon Lee, Luke Flewwelling, Madison Garibotti, Homira Osman, Arthur Cheng, Anthony Scimè, Christopher G.R. Perry, Kessen Patten, Natasha Chang, Rashmi Kothary, Jean-Philippe Leduc-Gaudet

**Affiliations:** 1 Department of Neurosciences, 5622 University of Montreal, Montreal, Canada; 2 University of Montreal Hospital Research Center (CRCHUM), Montreal, Canada; 3 Regenerative Medicine Program, 10055 The Ottawa Hospital Research Institute, Ottawa, Canada; 4 Muscle Health Research Centre, School of Kinesiology & Health Science, Faculty of Health, 7991 York University, Toronto, Canada; 5 459728 Muscular Dystrophy Canada, Toronto, Canada; 6 Department of Biology, York University, Toronto, Canada; 7 14850 Armand-Frappier Santé Biotechnologie Research Centre, Laval, Canada; 8 Goodman Cancer Institute, 5620 McGill University, Montréal, Canada; 9 Department of Biochemistry, Faculty of Medicine and Health Sciences, 5620 McGill University, Montréal, Canada; 10 Centre for Neuromuscular Disease, 6363 University of Ottawa, Ottawa, Canada; 11 Department of Medicine, University of Ottawa, Ottawa, Canada; 12 Groupe de Recherche en Signalisation Cellulaire, Département de Biologie Médicale, 14847 Université du Québec à Trois-Rivières, Trois-Rivières, Canada

**Keywords:** neuromuscular disorders, neuromuscular research, translational research, standardized protocols

## Abstract

The Neuromuscular Disease Network for Canada aims to accelerate research and improve care for individuals living with neuromuscular disorders by connecting basic scientists, clinicians, and trainees nationwide. As part of this mission, the network held its second Basic Research Summer School (May 7–8, 2025, York University, Toronto), integrating patient and caregiver perspectives with advanced scientific sessions and full-day methodological workshops. The program emphasized experimental rigor, reproducibility, and collaboration across research pillars, while launching the Basic Science Trainee Committee and Canada's first national, publicly accessible database of standard operating procedures for muscle research. This trainee-led, reproducibility-focused model of Summer School provides a transferable framework for building standardized preclinical capacity across international neuromuscular disorders research networks.

## Introduction

Neuromuscular diseases (NMDs) comprise a heterogeneous group of over 600 inherited and acquired disorders affecting skeletal muscle, peripheral nerves, neuromuscular junctions, or motor neurons.^1,2^ While individually rare, NMDs collectively represent a substantial health burden, leading to progressive muscle weakness, functional impairment, and, in many cases, premature mortality.^3^ Conservative prevalence estimates suggest that up to 90 per 100,000 individuals are affected worldwide, although underdiagnosis remains common.^4–6^ Advances in genetic diagnostics, including next-generation sequencing, have accelerated the discovery of causative genes and enabled the development of novel targeted therapies.^7,8^ However, translating fundamental discoveries into effective treatments remains challenging due to disease complexity, variability in clinical presentation, and limitations in preclinical-to-clinical transferability.^9^

A major obstacle to translational progress in NMD research is the persistent lack of reproducibility in preclinical models. Differences in animal handling, functional assays, and histological protocols across laboratories lead to inconsistent data and poor cross-study comparability.^9–11^ To overcome these limitations, the neuromuscular field increasingly recognizes the need for coordinated, standardized, and transparently validated methodologies developed through collaboration between basic scientists, clinicians, patient partners, and industry.

Founded in 2020, the Neuromuscular Disease Network for Canada (NMD4C) unites more than 800 members across three pillars: preclinical science, clinical research, and education, to accelerate therapeutic development and promote standardized care for people living with NMDs.^12^ A core objective of the network is to strengthen national research capacity by training the next generation of scientists. Recognizing that sustainable progress depends on methodological rigor, NMD4C launched the Basic Research Summer School to provide immersive, skills-based education that links advanced experimental training with patient-centered perspectives, underscoring the societal impact of translational research.

Building on the success of its inaugural edition, the second NMD4C Basic Research Summer School was held on May 7–8, 2025, at York University in Toronto. The program introduced the trainee-led Basic Science Trainee Committee (BSTC), responsible for developing Canada's first national, publicly accessible database of standard operating procedures (SOPs) for muscle research, an initiative launched in early 2025 to address reproducibility gaps in preclinical studies. The two-day event gathered 32 trainees from across Canada, together with patient, industry, and institutional representatives, to foster collaboration and shared methodological standards. By combining patient engagement, scientific exchange, and immersive technical training, the Summer School strengthened methodological rigor and collaboration across the neuromuscular research community. The NMD4C model, designed around methodological rigor and trainee leadership, offers a scalable approach that could inform similar initiatives internationally.

## Setting the stage for translational research

The Summer School opened with a session establishing both the scientific and cultural direction of the event. Opening remarks by Dr Chris Perry (York University) outlined NMD4C's mission to accelerate neuromuscular research and improve care through coordination across its preclinical, clinical, and educational pillars.^12^ The introduction emphasized that sustained progress depends not only on scientific innovation but also on aligning laboratory practice with the lived experiences of people affected by NMDs and supporting long-term career development for trainees.

This perspective framed the launch of the Basic Science Trainee Committee (BSTC), led by Dr Jean-Philippe Leduc-Gaudet (chair), Dr Emma Sutton (co-chair), and Adrien Rihoux (secretary). Acting as a bridge between trainees and network leadership, the BSTC spearheads the creation of Canada's first publicly accessible SOP database for muscle research—an initiative designed to reduce methodological variability and strengthen reproducibility in preclinical studies. Together, these efforts define the School's dual focus: advancing scientific rigor while fostering collaboration and inclusivity across the neuromuscular research community.

*Patient perspectives:* A core goal of the Summer School was to connect trainees with the lived experiences of individuals affected by neuromuscular disorders. The perspective of Meghan Hines, an advocate living with an NMD, was used to illustrate how research priorities should extend beyond curative therapies to address daily quality-of-life challenges and transparent communication between patients and clinicians. A caregiver viewpoint, presented by Terrence Ho, highlighted persistent gaps in adult care despite advances in pediatric management for Duchenne muscular dystrophy. Together, these contributions grounded the scientific program in patient-centred priorities. They emphasized that translational research gains relevance only when methodological rigor and reproducibility serve the goal of improving real outcomes for people living with NMDs.

*Scientific presentations:* Scientific sessions built on the patient discussions, translating real-world priorities into laboratory context. Presentations from Dr Lisa Hoffman (Western University), Dr Anthony Scimè (York University), Dr Dean Betts (Western University), Dr Charles Kassardjian (University of Toronto), and Dr Jean-Philippe Leduc-Gaudet (Université du Québec à Trois-Rivières) addressed complementary aspects of muscle biology and disease mechanisms. Topics ranged from vascular dysfunction in Duchenne muscular dystrophy^13,14^and metabolic regulation of muscle and stem-cell function,^15^ to variability in corticosteroid management in myasthenia gravis^16,17^ and reproducible histological analysis of muscle tissue.^18–21^ Across these talks, the shared message was clear: reproducibility and methodological precision are indispensable for bridging preclinical research with meaningful clinical translation. This thematic focus set the stage for subsequent sessions on industry collaboration and training.

*Industry engagement and career development:* After the scientific presentations, the program moved to explore how new discoveries and methodological advances can be applied in practice. An integrated 3-in-1 testing system for rodent muscle physiology enabling *in vivo*, *in situ*, and *in vitro* assessments within a single platform, was presented by Katherine Anthaide (Aurora Scientific Inc.). This technology illustrates how industry partnerships can accelerate translation by providing researchers with validated, reproducible tools. The day concluded with a career development roundtable, where panelists from academia, industry, non-profit, and biotechnology, including Dr Youn Hee Cho (BenchSci), Dr Jean-Philippe Leduc-Gaudet (Université du Québec à Trois-Rivières), Dr Homira Osman (Muscular Dystrophy Canada), and Chris Rand (Aurora Scientific), shared their professional trajectories. Discussions were interactive, with trainees actively engaging with the panel through questions and exchanges about their own career considerations. Panelists highlighted the diverse pathways available, the transferable skills valued across sectors, and strategies for aligning personal research interests with broader career goals. For many attendees, this two-way dialogue helped clarify how their work could generate impact both within and beyond academia.

## Hands-on workshops: putting translational science into practice

The second day of the summer school was devoted to full-day workshops that immersed participants in advanced methodologies central to neuromuscular research. Designed as a practical counterpart to the scientific discussions, these sessions combined expert instructions with active participation, allowing trainees to work through each stage of the techniques themselves. The goal was not only to introduce innovative tools and approaches but also to strengthen the technical standards and reproducibility essential for impactful research.

*Rodent Muscle Function Assessment:* The Rodent Muscle Function Assessment workshop, coordinated by Dr Arthur Cheng (York University), focused on establishing reliable and reproducible functional outcome measures in preclinical neuromuscular models. Participants conducted both *in vivo* and *in vitro* experiments to measure muscle force, fatigue, and recovery, applying standardized procedures under controlled conditions. The training emphasized sample integrity, calibration accuracy, and consistent protocol execution, core principles for generating comparable and reproducible data across laboratories.

*Myofiber Isolation & Analysis:* The Myofiber Isolation & Analysis session, led by Dr Anthony Scimè (York University), introduced trainees to *ex vivo* methods for studying muscle stem cells (MuSCs). The workshop demonstrated the single-myofiber isolation technique,^22,23^ which preserves MuSCs within their native niche to enable physiologically relevant analysis of activation, proliferation, and differentiation. Through this exercise, participants learned how standardized handling and preservation practices directly influence data quality and reproducibility, complementing the functional assays covered in the preceding session.

## Conclusions

The second NMD4C Basic Research Summer School brought together patient perspectives, scientific sessions, industry collaboration, and practical training to advance translational neuromuscular research. By connecting lived experience with methodological rigor, the event underscored how reproducibility and patient-centred priorities together enhance the impact of preclinical studies. Its key outcomes included hands-on training in functional and stem-cell assays and the establishment of the Basic Science Trainee Committee's national SOP database, an initiative designed to promote methodological standardization and sustainable research capacity. By integrating methodological rigor, patient engagement, and trainee leadership within a national SOP initiative, the NMD4C model establishes a scalable framework that could inform similar capacity-building efforts in neuromuscular research worldwide.
